# Increasing opportunities for movement in Nova Scotia, Canada: a community case study

**DOI:** 10.3389/fpubh.2026.1739584

**Published:** 2026-02-19

**Authors:** Emily Burke, Sara Kirk, Simran Bhamra, Stephanie Heath, Elaine Shelton, Maggie Locke, Sarah A. Moore

**Affiliations:** 1 School of Health and Human Performance, Faculty of Health, Dalhousie University, Halifax, NS, Canada; 2 Healthy Populations Institute, Dalhousie University, Halifax, NS, Canada; 3 Research Power Inc., London St. Halifax, NS, Canada; 4 Department of Communities, Culture, Heritage, and Tourism, Halifax, NS, Canada; 5 Department of Pediatrics, Faculty of Medicine, Dalhousie University, Halifax, NS, Canada

**Keywords:** movement, health, community-based, physical activity, rural, participatory evaluation

## Abstract

**Introduction:**

Rural and aging populations in Nova Scotia experience high burdens of chronic disease linked to insufficient physical activity and excessive sedentary time. *Communities on the Move* (CoM) is a four-year (2022–2026) community-wide initiative designed to increase opportunities for low-barrier, less-structured movement (e.g., walking, wheeling, cycling, gardening, etc.) through policy, built-and socia-environment actions and cross-sector partnerships.

**Objectives:**

To describe the CoM Phase 1 actions across four Nova Scotian communities, detail the participatory evaluation approach, and share early implementation insights.

**Methods:**

CoM applies a socio-ecological and participatory approach. Four communities were selected via a competitive application process assessing readiness and investments for active transportation. Communities received funding, communication support, and access to a multisectoral network to develop and implement action plans. A multi-phase, mixed-methods evaluation (Phase 1: Fall 2022-Spring 2023) tracked activities and outcomes.

**Results:**

Phase 1 findings show that three communities formed leadership teams, delivered social programs, improved infrastructure, and initiated a public engagement campaign to promote a culture of movement. One community experienced a delay in leadership formation due to staff leave; however, existing leaders and partners were leveraged to drive project actions. Feedback from communities shows early indicators that CoM has strengthened community capacity for cross-sector collaboration and resource mobilization.

**Conclusion:**

This community case study offers guidance for others to consider when implementing and evaluating community movement initiatives. Early findings align with existing evidence which suggest that pairing environmental changes with social supports and sustained partnerships to be feasible and promising for implementation potential in rural, aging contexts. Phase 1 evaluation concentrated on process outputs that establish the groundwork for future outcome assessments. Subsequent phases will build on these foundations to further examine changes in movement behaviors, participation, and community capacity.

## Introduction

Physical inactivity and sedentary behavior are major global health concerns, affecting over 27% of adults worldwide (1). In Canada, nearly half of adults (49%) do not meet recommended physical activity (PA) guidelines (2), and adults in Nova Scotia are no exception (3). This is a critical public health issue, as insufficient PA is strongly associated with chronic diseases such as cardiovascular disease, type 2 diabetes, and certain cancers, which contribute significantly to morbidity and mortality in Nova Scotia (4). For example, in 2023, Nova Scotia reported the highest rate of obesity in the country at 46% (5).

This community case study describes *Communities on the Move* (CoM), a provincial initiative designed to increase opportunities for daily movement through policy, built- and social-environment changes and cross-sector partnerships. Four communities were selected to participate in the project based on assessments of readiness. This study outlines CoM's first-year actions and participatory evaluation approach, offering early implementation insights to inform similar initiatives.

## Background and rationale

The impact of physical inactivity is compounded by Nova Scotia's aging and rural population. By 2030, more than one-quarter of Nova Scotians will be aged 65 or older (6), and about 40% of Nova Scotia's population resides in rural areas (7). Older adults are particularly vulnerable to the health risks of physical inactivity, including increased chronic disease burden and premature mortality (6). However, rural communities often face limited access to PA infrastructure and recreational facilities (8). Intervening to create more opportunities for movement among older adults, and rural residents, could reduce disease prevalence, lowering healthcare costs and resources associated with chronic disease management in Nova Scotia (9).

To address these challenges, the Province of Nova Scotia has introduced several policies and action plans to promote healthy movement, including the *Let's Get Moving Nova Scotia* initiative, which aligns with the federal approach to increase PA and reduce sedentary time (3, 10). These efforts emphasize low barrier, less structured movements that can be easily integrated throughout the day, including walking, wheeling, active transportation (AT) and unstructured play (3). Increasing opportunities for daily movement, offers broad benefits, not limited to improved health outcomes, enhanced social cohesion, reduced commuting costs, and environmental gains through decreased air pollution (10, 11). AT refers to any non-motorized transportation in which one powers themselves from one place to another, this may include walking, wheeling, or cycling (12). AT infrastructure also supports the mobility and independence of populations that experience barriers to automotive travel, such as older adults (13).

Community design and the built environment play a key role in shaping opportunities for movement. Automobile-centric planning has prioritized motorized transport over walkability, limiting infrastructure for AT and daily movement (13, 14). Features such as continuous sidewalks, safe crossings, and well-lit streets encourage walking and wheeling, while their absence creates barriers to AT (15, 16). In Nova Scotia, many rural communities lack adequate sidewalks and bike lanes, while urban areas often face safety concerns such as high-volume traffic (13, 18). Evidence suggests that combining built environment improvements (e.g., sidewalks, lighting, traffic calming) with social environment interventions (e.g., promotional programs) and supportive policies is more effective than implementing built environment approaches alone (17, 20). This underscores the need for multi-component, community-specific interventions to improve environments and promote everyday movement across Nova Scotia. Accordingly, community coalitions will be essential for maximizing resources, leveraging partnerships, and driving local action.

## Description of the case

CoM was launched in 2022 by Nova Scotia's Department of Communities, Culture, Tourism, and Heritage (CCTH) to strengthen community capacity for movement-friendly environments and initiatives. Four communities were selected to participate in the project based on a competitive application process that assessed readiness and existing plans and investments for AT infrastructure. The CCTH supports communities by providing funding, implementation support, and access to a multisectoral learning network. Each community develops annual actions plans integrating policy, infrastructure, and engagement strategies. A four-year participatory evaluation tracks implementation process (i.e., what is being done) and outcomes (what is being achieved).

## Settings

**Figure 1:**
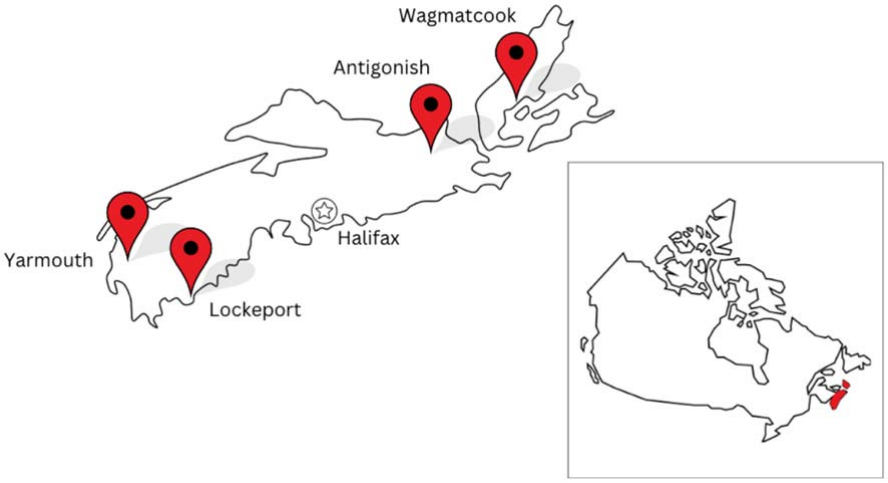
Map of CoM communities in Nova Scotia, Canada.

The four CoM communities include the Municipality of the County of Antigonish, the Town of Lockeport, the Municipality of the District and Town of Yarmouth (with coordination in the Town of Yarmouth), and the community of Wagmatcook First Nation. The CoM communities represent diverse geographic (Figure 1) and demographic contexts (Table 1), including rural and semi-urban areas with aging populations and varying levels of AT and PA infrastructure. Local leaderships teams comprise municipal/band staff, health professionals, recreation leaders, and community organizations.

**Table 1:** Community characteristics.

**Characteristic**	**County of Antigonish**	**Town of Lockeport**	**Municipality of the District of Yarmouth**	**Wagmatcook First Nation**
Area	1,456.42 km^2^	2.32 km^2^	2,121.64 km^2^	3.72km^2^
Population	20,129	476	24,947	691
Average age (years)	44.3	57.6	46.7	30.0
% of population women	51.64%	55.79%	51.58%	49.6%
Median total household income	$71,500	$50,800	$68,500	$56,800

Data from Statistics Canada, “Census profile: 2021 Census of Population” (43).

Antigonish County is a rural region located along the province's Northumberland shore. Much of the County's population is dispersed, with a population density of 13.8/km^2^ (19). In 2021, 93% of Antigonish County's population identified as white with the largest visible minority populations being reported as Black (2%), South Asian (1%), and Filipino (1%). The Town of Lockeport is located within Shelburne County on the South Shore of the province. The majority of the population lives on a small rural peninsula, resulting in a population density of 205.4/km^2^ (19). In 2021, 100% of the Town of Lockeport's population identified as white, with cultural origins including Irish (23%), Scottish (22%), and German (16%) (19). The Municipality of the District of Yarmouth is located at the Southwestern tip of the province. Similar to Antigonish, much of the population lives in rural areas, with an overall population density of 17.2/km^2^ (19). In 2021, 98% of the district's population identified as white, with the largest visible minority populations being reported as Black (1%), South Asian (0.3%), Filipino (0.2%), and Korean (0.2%) (19). Lastly, Wagmatcook (Waq-mit-kuk) is a First Nation reserve located on Cape Breton Island (also known as Unama'ki). Wagmatcook was the first permanent settlement of the Mi'kmaq Nation in the province (21). The community is bilingual, both English and Mi'kmaq languages are spoken. In 2021, Wagmatcook First Nation reported a population of 691 and population density of 185.8/km^2^ with 95% of the band's population identifying with Mi'kmaq, First Nations, or North American Indigenous ethnic or cultural origins (20).

## Methods

CoM uses a socio-ecological framework and community-based participatory approach to guide multi-level interventions. Action plans, co-designed by local leadership teams and partners, integrate policy, built environment, and social strategies. Implementation occurs in four phases: planning, delivery, scaling, and sustainability (2022–2026). Evaluation employs mixed methods, assessing process indicators (e.g., leadership formation, infrastructure changes) and outcome measures (e.g., PA, program engagement).

### Program overview

The CoM initiative aims to increase community-wide engagement in daily movement through multicomponent interventions across four Nova Scotian communities. Action plans, co-designed by local leadership teams with public-sector and academic partners, integrated policy, built environment, and social environment strategies, guided by principles of equity and access. Plans aligned with five objectives of the CCTH's Municipal/Mi'kmaw Physical Activity Leadership Program: (1) social supports for walking, (2) physical environment supports for walking, (3) social supports for other less-structured movement, (4) physical environment supports for other less-structured movement, and (5) policies to support movement (44). Priority populations included older adults and equity-denied groups with limited access to movement opportunities.

Sustainability was supported through a community-based participatory approach involving shared decision-making, distributed responsibilities, tailored interventions, and capacity building. Community leadership teams developed annual action plans and guided implementation. Academic partners co-created the logic model and evaluation framework provided training, and led evaluation activities and participatory interpretation. Public-sector partners provided funding and coordination, while private and community organizations delivered program components and in-kind supports. One key example is *Make Your Move* (MYM), a public engagement campaign launched by the Healthy Tomorrow Foundation to encourage daily movement (22). Adapted locally, MYM served as a central social environment component of CoM. Partnerships among government, academia, and communities were critical for leveraging resources and driving action for this initiative.

### Theoretical framework

CoM is guided by a socio-ecological framework, which recognizes that individual movement behaviors are shaped by multiple levels of influence, including social and environmental factors (23). This framework is widely used in health promotion practice, as it supports comprehensive multi-level interventions to address complex health issues (24). Accordingly, CoM combined built-environment enhancements (e.g., benches, sidewalk extensions) with social supports (e.g., awareness campaigns) and policies (e.g., workplace movement breaks) to reduce barriers and promote daily movement. This approach aligns with provincial and federal strategies that emphasize supportive environments, partnerships, and multi-component interventions to increase participation in PA and movement (3, 10).

### Implementation

CoM was implemented in four consecutive phases. Phase 1 (April 2022–March 2023) focused on start-up and planning, including leadership team formation, development and initial implementation of community-wide movement plans, initiation of MYM engagement, and finalization of the evaluation framework and logic model. Phase 2 (April 2023–March 2024) advanced implementation through the continued delivery of social-support programs, infrastructure enhancements such as benches and sidewalk extensions, and expansion of partnerships and communication strategies. Phase 3 (April 2024–March 2025) emphasized scaling and integration, including further built-environment changes, institutionalizing supportive policies, and strengthening coalition capacity. Phase 4 (April 2025–March 2026) focuses on sustainability and monitoring, with formal evaluation activities concluding in March 2026.

CoM embedded collaboration throughout implementation by operationalizing community-based participatory principles in practice. Communities were equal partners, co-developing action plans for each phase and the evaluation framework to ensure alignment with local priorities and contexts. Decision-making was shared among municipal and band staff, funders, and academic partners, with leadership teams guiding priority setting through consultations and baseline assessments. Community members also contributed to data collection for the program's evaluation and actively participated in interpreting findings alongside academic partners. To support cyclical learning and adaptability, all evaluation findings and knowledge generated were shared back with local leadership teams. Knowledge products were tailored for each community to ensure usability and practicality, empowering those involved. This approach fostered ownership, cultural relevance, and sustainability while strengthening trust and capacity within each community.

### Evaluation design

The CoM evaluation uses a mixed-methods, participatory approach guided by the project's logic model and evaluation framework (see logic model in Appendix A). The framework integrates process and outcome measures across four components: partnerships and leadership, community capacity, engagement and awareness, and natural and built environments. The research sample includes leadership team members, program participants, and residents from the four participating communities. Leadership team members were purposively selected based on their roles in planning and implementation. Broader community engagement will use convenience sampling for surveys and accelerometry, complemented by targeted outreach to equity-denied groups. Phase 1 primarily assessed process outputs, such as leadership team formation, communication strategies, enhancements to programming, and early infrastructure improvements. These outputs establish conditions for later outcome evaluations, which will examine effectiveness and community-wide behavior changes, including PA and sedentary time, cross-sector collaboration, community capacity, and culture of movement. Phase 1 data collection methods included document review, interviews with leadership team members, and activity tracking forms, complemented by baseline infrastructure usage counts.

Future phases will incorporate surveys, accelerometry, repeat infrastructure usage counts, and further qualitative inquiry to deepen understanding of implementation effectiveness and outcomes across priority populations. Infrastructure usage counts will occur each Fall at selected sites (3–4 sites per community) in which AT infrastructure plans to be improved. These counts will be collected over three days (Thursday, Friday, and Sunday) for eight hours a day (7 a.m.−9 a.m., 11 a.m.−1 p.m., and 3 p.m.−7 p.m.), following a consistent schedule to enable comparisons across time. Evaluations are scheduled annually, with baseline data collected in Phase 1 and follow-up assessments in Phases 2–4. Changes in PA and sedentary time will be assessed using self-reported surveys and accelerometry. Community capacity (i.e., the ability of leadership teams to mobilize resources and deliver movement initiatives) will be assessed through indicators such as leadership diversity and training, co-funding security, and policy changes. Cross-sector collaboration will be measured by the number and diversity of sectors represented in leadership teams and partnerships, as well as the frequency of joint planning activities. Culture of movement will be evaluated through engagement metrics, including infrastructure usage counts, participation in events, and MYM campaign reach, complemented by qualitative feedback on community norms and attitudes toward daily movement.

The CoM evaluation was exempt from REB review, as outlined in TCPS article 2.5 (45). Still, the CoM evaluation team adhered to all recommended ethical guidelines. Informed consent procedures were followed for interviews/focus groups.

Community-based participation was integral to the evaluation design and implementation. Evaluation leads from Dalhousie University and Research Power Inc. collaborated with community leadership and CCTH to co-develop the logic model and evaluation framework, link activities to anticipated outcomes, and identify indicators for process and outcome measures. Leadership teams contributed to baseline assessments, document reviews, and interpretation of findings, and community members were trained and supported to participate in data collection (e.g., activity tracking and infrastructures usage counts). This collaborative approach ensures evaluation results are meaningful and informs iterative refinements to action plans.

This paper focuses on implementation feasibility and early process indicators and outputs from Phase 1. As the subsequent phases of CoM are implemented, future work will aim to evaluate the effectiveness of the program and address community behavioral change.

## Results

Across all four communities, early efforts centered on leadership development, action planning, and foundational activities aligned with provincial objectives. A brief overview of Phase 1 community actions mapped to evaluation components are shown in Table 2. Infrastructure usage counts were collected as part of baseline assessments but are not reported with Phase 1 results because exposure time to new infrastructure and social changes was insufficient to produce meaningful differences. These baseline counts will serve as reference points for comparison in subsequent phases and will be reported upon the final evaluation in 2026.

**Table 2:** Phase 1 actions mapped to evaluation components.

**Evaluation component**	**Key process indicators**	**Examples of phase 1 actions across communities**
Partnerships & leadership	Development of local leadership groups; cross-sectoral engagement and partnerships	**Antigonish:** Formed 19-member leadership team; engaged schools, housing, health, business sectors. **Lockeport:** Leadership group (8 members); partnerships with schools and recreation committee. **Yarmouth:** Leadership committee (eight members); partnerships for active transportation projects and engagement with local First Nations Band to promote collaboration. **Wagmatcook:** Leveraged Trails Committee; partnerships with provincial organizations
Community capacity	Enhancements to programming; leadership training; projects for equity-denied groups	**Antigonish:** Developed annual action plan; identified gaps in workplace engagement; walk leader training provided for free to community and leadership team members. **Lockeport:** Developed annual action plan; walk leader training; winter walking initiatives; dementia-friendly signage. **Yarmouth:** Development annual action plan; supported cross-sector planning of large-scale active transportation projects; walk leader training. **Wagmatcook:** Walk leader training; re-established walking group; supported pop-up bike hubs
Community engagement & awareness	Communication strategies; engagement activities; social-environment improvements	**Antigonish:** Make Your Move branding; community social groups planned ‘monthly movement connectors'; spot lighted community movement champions through social media. **Lockeport:** Multigenerational walking event; Make Your Move banners and branding; increased social media presence. **Yarmouth:** Virtual walking challenges; Make Your Move Seniors Expo; seasonal walking campaigns. **Wagmatcook:** Mi'kmaw translation of Make Your Move branding
Natural & built environments	Supports to enhance infrastructure; physical-environment improvements	**Antigonish:** Sidewalk extensions; benches; active transportation corridor projects. **Lockeport:** Multi-use pathway project; benches; wayfinding signage; and lighting assessment. **Yarmouth:** Investments in multi-use trails; sidewalk and bike lane additions; park playboxes installed. **Wagmatcook:** Active transportation and cycling network; pop-up bike hubs

Actions listed in the table represent Phase 1 process outputs. Outcomes (e.g., changes in PA levels, culture shifts, etc.) will be assessed in Phases 2–4.

In Phase 1 all communities formed or strengthened cross-sector leadership teams to guide CoM implementation. Antigonish established a 19-member leadership group representing recreation, health, education, housing, business, and climate sectors. Lockeport and Yarmouth created leadership committees with municipal staff, educators, business leaders, and, community representatives. Wagmatcook's Phase 1 implementation was delayed due to staff leave, which affected the leadership team formation and slowed planning. Mitigation strategies included appointing an interim project coordinator, leveraging the Band's existing Trails Committee and partnerships with provincial organizations to advance infrastructure projects and culturally relevant engagement activities.

Communities developed annual CoM action plans to guide environmental and policy interventions (excluding Wagmatcook, for reasons noted above). Plans were tailored to fit current community contexts, leveraging existing resources and partnerships, and incorporated the voices of community members. Community leaders assisted with baseline assessments of local built-and social environments, leadership and partnerships, and policy and planning for daily movement. Assessments were conducted through consultations with residents and stakeholders, review of municipal and organizational documents, and on-the-ground observations. Based on these assessments, each leadership team identified priority areas for support. For example, Lockeport recommended improving multi-use pathways and improving safety, while Antigonish emphasized trail connectivity and Yarmouth prioritized social engagement activities and movement networks. These recommendations informed the design of community action plans and guided resource allocation. Additionally, the CCTH provided communities with regular access to a multi-sectoral learning group (including CoM leadership team members, academic, healthcare, and public-sector personal, and a consultant from the Albert Lea Blue Zone project (25), to support the development of action plans and offer insights and education on movement-promoting interventions.

Capacity-building was a key focus. Across communities, 17 Nova Scotia Walk Leaders were trained, resulting in new walking groups and strengthened leadership for existing groups (26). Lockeport hosted a multigenerational walking event with over 130 participants, including grandparents, students, and local school staff. Yarmouth launched the ‘Active Soles' walking group for older adults and organized multiple walking challenges, while Antigonish organized monthly movement connectors and a MYM community celebration event featuring bike rodeos and e-bike testing. Wagmatcook established local walking groups and hosted a pop-up bike hub in partnership with a provincial cycling organization to train community members on bike maintenance and safety. Additional training included HopOn cycling instructor certification (5 instructors) (27) and Nova Scotia Active Smarter Kids training for 25 educators in Lockeport (28).

Infrastructure improvements were notable across sites. Yarmouth supported large-scale AT projects, including the Lake Milo AT project, sidewalk additions in three locations, and new bike lanes; Lockeport paved multi-use pathways, installed benches, and dementia-friendly wayfinding signage; Antigonish expanded its community AT network, prioritizing areas with high numbers of older adults, and installed AT signage supported by provincial and federal funding; and Wagmatcook initiated Blue Route, a cycling route network (29). Across communities, bike initiatives included rodeos, group rides, and loan programs, supported by infrastructure such as bike storage barns and racks. Social supports extended beyond walking, with Antigonish offering subsidized outdoor recreation programs and free skating and swimming sessions (all sessions reached capacity), and Yarmouth introducing family play sessions and community scavenger hunts.

Branding and communication strategies under the MYM campaign were initiated in all communities, with Wagmatcook translating materials into Mi'kmaw to promote cultural relevance. Engagement activities included community walks, seasonal AT campaigns, and movement expos, supported by social media and promotional materials. For example, Antigonish showcased local community movement leaders on social media, with individuals representing a broad range of age groups and abilities, including older adults. Leadership feedback emphasized the importance of consistent messaging and branding to foster awareness and normalize daily movement.

Findings from Phase 1 revealed several facilitators, including strong municipal and provincial partnerships, prior AT work, and the availability of provincial MYM branding and resources to support local adaptation. Key challenges included engaging workplaces, health settings, reaching equity-denied populations; limited capacity for sustained marketing in smaller communities; and infrastructure delays due to resource constraints. Overall, community leadership teams reported enhanced capacity-for resource mobilization through perceived strengthened collaboration across sectors, which supported early project implementation. They also highlighted the need for clear role definition and flexible strategies to adapt to evolving community contexts.

## Discussion

### Practical implications

This paper illustrates a community case study that addresses increased movement through coordinated planning in the built and social environment and policy across four communities in Nova Scotia, Canada. The project elements may serve as a framework for health promoters and leaders who wish to develop and implement comprehensive interventions aimed at creating healthier communities through increasing opportunities for simple movement. Long-term implementation is required to fully actualize the potential of community-based participatory interventions (30). However, considerable evidence supports the cost-effectiveness of population- and community-wide physical and social environment interventions for the promotion of PA (31, 32).

Previous interventions like CoM have reported promising results in relation to community-wide healthy movement behaviours and community wellbeing; for example, the 2009 Albert Lea Blue Zone project resulted in increased AT and PA among community residents, as well as increased sense of community spirit and sense of purpose among older adults (33). In addition, Voorstad on the Move, a Netherlands-based community health promotion program grounded in the socioecological perspective, resulted in increased healthy behaviours, social connection, and meaningfulness among active community participants (34).

It is important to consider the context in which you are implementing an intervention. The CoM project aims to increase PA and AT in rural Nova Scotian communities, with a large older adult population. Research has shown that the physical environment of one's community or neighborhood can impact members' PA participation through increasing walkability within community infrastructure (35). Furthermore, older adults residing in rural communities are less likely to be physically active compared to those living in urban settings due to lower walkability and AT infrastructure (36, 37). The CoM project has considered this context, knowing the importance of promoting PA and movement through AT for those living in rural communities and those within equity-denied groups, who may experience greater barriers to PA. Therefore, based on the literature we anticipate that the CoM project will have positive impacts on individual and population-level participation in PA and healthy movement behaviors across the four communities. Like the Albert Lea Blue Zone and Voorstad on the Move projects, we expect the CoM project to facilitate improved social environments for movement (e.g., community spirt and social connection), as community members network and connect when engaging in movement (33, 34). Changing the culture of movement within communities is not an easy feat. Yet, by partnering with community members and leaders, this project will work toward sustainable improvements in AT infrastructure, promoting a healthy, active lifestyle.

Through creating increased and improved spaces and places for simple movement, communities can encourage greater participation, leading to positive impacts on health and well-being across settings and populations, with the potential to change community members' daily movement behaviors (8, 38). Although context is an important factor for success in implementing community programs such as this one, the strategies used in CoM such as building community relationships and setting community goals can be used widely across communities to ensure programs are addressing specific community needs.

### Lessons learned

Over the first year of the CoM project's implementation, valuable lessons have been learned. First, in order to meet the needs and understand the local context of each community and ensure buy in and uptake of the approach and evaluation, the CoM project emphasized building meaningful relationships with community members and organizations. Through understanding the different needs of each community, the CoM team worked collaboratively with communities to set priorities and goals that were sustainable and unique to their context. Examples of implementation to achieve community goals, promoting PA and AT, include infrastructure change, program development and sustainment, and community outreach, all of which will be implemented and assessed in the following phases of the CoM project.

The second lesson learned in the first phase of the CoM project was that each community's CoM leadership team had varying levels of knowledge and resources to support movement across settings and equity-denied populations. This meant different approaches were needed to support each community based on their level of readiness (e.g., hands-on learning, one on one coaching, etc.). Level of readiness also impacted how quickly community action plans could be developed and when the first phase of data collection could begin. For example, Antigonish was able to build a large leadership team representing diverse sectors, while Lockeport and Yarmouth were able to build a leadership team, which had slightly less diversity and a smaller number of participants. As these three communities had support from community members, organizations, and government departments, they were ready for baseline data collection to occur during Phase 1. The community of Wagmatcook First Nations faced barriers, which delayed their readiness for data collection as staff went on leave, impacting the formation of their leadership team, yet they will complete baseline data collection in Phase 2 of the project.

Another challenge in Antigonish illustrated the importance of aligning activities with community capacity. Monthly social groups designed to promote movement could not be sustained due to scheduling conflicts among volunteer facilitators and limited availability of shared community spaces. This highlighted the need for flexible program formats and early engagement of dedicated community champions. Overall, these lessons emphasize the importance of adapting implementation plans to each community's readiness, cultural context, and available resources while balancing project evaluation needs.

### Expected future impacts

Through collaboration of researchers from Dalhousie University and Research Power Inc., community leadership groups, and CCTH we co-developed the logic model and evaluation framework that have guided the implementation of Phase 1 and will guide the following future phases of the CoM project. Furthermore, based on the results of similar PA community program we expect that through building connections with communities and helping to set sustainable goals to increase participation in AT within these communities, we will not only see an increase in PA amongst community members, but that this increase in PA will be sustainable after the completion of the project, as we expect there to be a positive shift within the culture of movements within these communities. Increasing PA and the culture of movement within communities may help to support community members in meeting the Canadian 24-h movement guidelines, ultimately increasing their physical and mental health and wellbeing (39). Outside of increasing AT and PA in the communities, AT is also closely related to environmental impacts, reducing greenhouse emissions and air pollution (40). Therefore, we expect that the community connections and relationships which have been established in Phase 1 will lead to positive physical and mental health outcomes for community members as well as positively contributing to environmental sustainability.

### Strengths and limitations

CoM is a collaborative and comprehensive approach to promoting healthy movement behaviours in Nova Scotia, exemplifying the potential impact of coordinated efforts and partnerships between government, academia, and local communities. The design of the CoM project and evaluation enables engagement from all partners and aims to strengthen community networks, capacity, and awareness for PA and AT initiatives. In addition, the community-based participatory approach enabled flexibility regarding the scale and timeline of project interventions and actions across all communities. This allowed the project to be tailored for each CoM community's level of readiness and ensured CoM leadership teams stayed engaged through all project stages. Community-based participatory approaches promote sustainability by involving partners, distributing responsibilities, tailoring interventions to end-users' needs, and improving community competencies (30). Such an approach incorporates the knowledge and values of all project partners (41). The participatory mixed-method, multi-phase evaluation offers a comprehensive assessment of CoM's process and outcomes measures, ensuring responsive project adaptation, knowledge mobilization, and strengthened organizational capacity for planning and evaluation within the four communities. The integration of community voice and transdisciplinary participation into the CoM project recognizes the complex social contexts of each community, enhancing strategic planning and structural support for PA and AT interventions (42). The project addresses physical inactivity and sedentary behaviours using a socio-ecological lens, takes a holistic approach to movement interventions, and incorporates the participation of community members, organizations, and networks to empower priority populations and enhance health equity.

Still, there are conceptual and methodological limitations of the CoM project to consider. For instance, the number of rural, low-density populations in Nova Scotia can make built environment interventions for movements such as walking and cycling challenging to meet the needs of communities unless it covers large areas. Additionally, the cost of the project's interventions, maintenance of project activities, and the time-consuming nature of participatory action and evaluation could act as major challenges for the project's future sustainability and the generalizability of the project's success.

## Conclusion

This paper presents a case study involving four communities that are engaged in initiatives to enhance movement, aiming to reduce the health risks associated with sedentary behavior and inactivity. This project highlights the need for safe and supportive built and social environments to encourage increased engagement in healthy movement behaviours at the population level. Our work demonstrates the potential of community-based participatory interventions and evaluations as communities have perceived positive strengthening in stakeholder engagement, community capacity, and movement networks that they feel empower priority populations and enhance health equity. Drawing insights from the first-year evaluation of CoM, we recommend health promoters assess community readiness, leverage existing resources, and encourage cross-sectoral engagement, collaboration, and flexibility in developing community-based interventions for PA and AT. This strategic approach is crucial for ensuring project success and long-term sustainability. Furthermore, we advocate for future interventions in PA and AT to adopt a socio-ecological approach, addressing built and social environments and policy. This comprehensive approach should prioritize community voices and foster collaborative partnerships among government, academia, and local communities. Such partnerships are essential for addressing existing social, environmental, and cultural factors that may act as barriers to movement across diverse populations and settings.

## Figures and Tables

**Appendix A:**
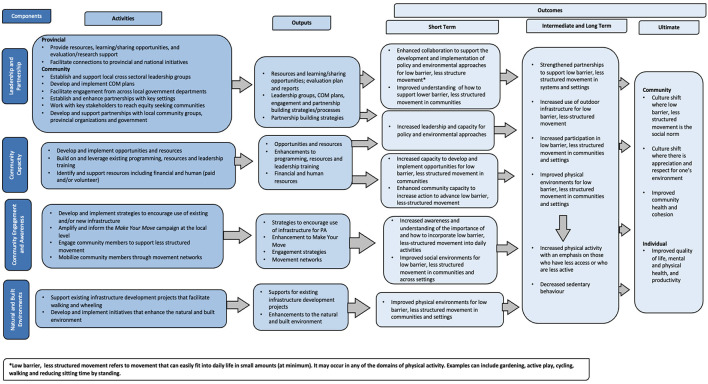
CoM logic model.

## Data Availability

The data supporting the conclusions of this article were provided by permission of the Province of Nova Scotia's Department of Communities, Culture, Tourism, and Heritage (CCTH). Data are available from the corresponding author upon reasonable request with permission of the CCTH.
